# Global gene expression profiling of pancreatic islets in mice during streptozotocin-induced β-cell damage and pancreatic *Glp-1* gene therapy

**DOI:** 10.1242/dmm.012591

**Published:** 2013-07-04

**Authors:** Jason M. Tonne, Toshie Sakuma, Michael C. Deeds, Miguel Munoz-Gomez, Michael A. Barry, Yogish C. Kudva, Yasuhiro Ikeda

**Affiliations:** 1 Department of Molecular Medicine, Mayo Clinic, Rochester, MN 55905, USA; 2 Human Cell Therapy, Department of Laboratory Medicine/Pathology, Mayo Clinic, Rochester, MN 55905, USA; 3 Department of Infectious Diseases, Mayo Clinic, Rochester, MN 55905, USA; 4 Division of Endocrinology, Mayo Clinic, Rochester, MN 55905, USA

## Abstract

Streptozotocin (STZ), a glucosamine-nitrosourea compound, has potent genotoxic effects on pancreatic β-cells and is frequently used to induce diabetes in experimental animals. Glucagon-like peptide-1 (GLP-1) has β-cell protective effects and is known to preserve β-cells from STZ treatment. In this study, we analyzed the mechanisms of STZ-induced diabetes and GLP-1-mediated β-cell protection in STZ-treated mice. At 1 week after multiple low-dose STZ administrations, pancreatic β-cells showed impaired insulin expression, while maintaining expression of nuclear Nkx6.1. This was accompanied by significant upregulation of p53-responsive genes in islets, including a mediator of cell cycle arrest, *p21* (also known as *Waf1* and *Cip1*). STZ treatment also suppressed expression of a wide range of genes linked with key β-cell functions or diabetes development, such as *G6pc2*, *Slc2a2* (*Glut2*), *Slc30a8*, *Neurod1*, *Ucn3*, *Gad1*, *Isl1*, *Foxa2*, *Vdr*, *Pdx1*, *Fkbp1b* and *Abcc8*, suggesting global β-cell defects in STZ-treated islets. The *Tmem229B*, *Prss53* and *Ttc28* genes were highly expressed in untreated islets and strongly suppressed by STZ, suggesting their potential roles in β-cell function. When a pancreas-targeted adeno-associated virus (AAV) vector was employed for long-term *Glp-1* gene delivery, pancreatic GLP-1 expression protected mice from STZ-induced diabetes through preservation of the β-cell mass. Despite its potent β-cell protective effects, however, pancreatic GLP-1 overexpression showed limited effects on the global gene expression profiles in the islets. Network analysis identified the programmed-cell-death-associated pathways as the most relevant network in *Glp-1* gene therapy. Upon pancreatic GLP-1 expression, upregulation of *Cxcl13* and *Nptx2* was observed in STZ-damaged islets, but not in untreated normal islets. Given the pro-β-cell-survival effects of *Cxcl12* (*Sdf-1*) in inducing GLP-1 production in α-cells, pancreatic GLP-1-mediated *Cxcl13* induction might also play a crucial role in maintaining the integrity of β-cells in damaged islets.

## INTRODUCTION

Streptozotocin (STZ) is a monofunctional nitrosourea derivative that was first derived from *Streptomyces achromogenes*. STZ is a potent alkylating agent, known as an alkylnitrosourea, that directly methylates DNA (Randerath et al., 1981). STZ-induced DNA damage is characterized by N7-methylguanine (N7-MeG): more than 70% of DNA methylation occurs at the N7 position of guanine (Capucci et al., 1995). In addition to covalent adducts (N7-MeG), STZ also induces DNA strand breaks in rat β-cells (LeDoux et al., 1986). Because of its selective toxic effects on pancreatic β-cells, STZ is frequently used to induce diabetes mellitus in experimental animals. To induce insulin-dependent diabetes, STZ is typically given through a single intravenous administration or through multiple low-dose intraperitoneal administrations (Deeds et al., 2011). Despite its extensive use in diabetes research, however, the influence of STZ treatment on pancreatic β-cells, especially on the global gene expression profile *in vivo*, remains elusive.

Proglucagon is cleaved to glucagon by prohormone convertase 2 (PC2) in α-cells, whereas glucagon-like peptide-1 (GLP-1) is synthesized when proglucagon is cleaved by PC1 in intestinal L cells. GLP-1 acts as an incretin hormone, which induces the stimulation of glucose-responsive insulin secretion and inhibition of glucagon secretion. GLP-1 also inhibits β-cell apoptosis while stimulating the proliferation of β-cells (Farilla et al., 2003). Despite its β-cell-trophic activities, the use of GLP-1 for clinical applications has been limited because of its short *in vivo* half-life, due to rapid degradation by the enzyme dipeptidyl peptidase-4 (DPP-4) (Mentlein et al., 1993). Several strategies have been used to accomplish sustained GLP-1 receptor activation, including DPP-4 inhibitors and GLP-1 receptor agonists that are resistant to DPP-4 degradation. Those drugs have gained widespread use for type 2 diabetes because of the demonstrated efficacy with low risk of hypoglycemia. Another strategy to overcome the short half-life of GLP-1 is through gene delivery. A single systemic administration of a *Glp-1*-expression plasmid can increase insulin secretion and decrease blood glucose levels for 2 weeks in insulin-resistant diet-induced obese mice (Choi et al., 2005). Adeno-associated virus (AAV)-vector-mediated delivery of a GLP-1-encoding sequence has also been shown to ameliorate STZ-induced hyperglycemia in mice for 5 weeks (Riedel et al., 2010).

GLP-1 is a well-characterized β-cell-trophic factor that also has insulinotropic and β-cell-protective properties. The regenerating islet-derived protein 3 (REG3) family proteins have been implicated in a range of physiological processes, including acting as a β-cell-trophic factors. For instance, islet neogenesis associated protein (INGAP), a member of the REG3 family proteins, is reported to reverse STZ-induced hyperglycemia through islet neogenesis (Rafaeloff et al., 1997; Gold et al., 1998; Rosenberg et al., 2004). In this study, to achieve maximum β-cell-trophic activities, we developed an AAV9-based β-cell-targeted gene-transfer system and delivered an artificial REG3B–GLP-1 fusion protein. This design allows overexpression of GLP-1, independent of proglucagon expression, as well as of REG3B to enhance islet growth and functionality. Although STZ and GLP-1 have been broadly used in diabetes research and therapy, the underlying mechanisms of STZ-induced hyperglycemia or GLP-1-mediated β-cell protection, especially in their influences on global gene expression profiles of pancreatic islets, remain elusive. Here, we analyzed the changes in pancreatic islets shortly after multiple low-dose STZ administrations, with or without prior pancreas-targeted *Reg3b–Glp-1* gene therapy. Our results demonstrate strong induction of p53-responsive genes and suppression of diabetes-related genes upon short-term low-dose STZ treatment. Pancreas-targeted REG3B–GLP-1 overexpression preserved the β-cell mass and protected mice from STZ-induced diabetes for 2 months. Unexpectedly, *Reg3b–Glp-1* gene therapy did not strongly affect STZ-imposed changes in global gene expression. Instead, pancreatic REG3B–GLP-1 expression suppressed the apoptosis pathway, and induced selected genes in STZ-damaged islets.

**TRANSLATIONAL IMPACT**

**Clinical issue**

Diabetes mellitus is increasing in an epidemic fashion worldwide; the number of affected adults is projected to be as high as 440 million by 2030. Thus, it is crucial that novel therapies are developed to treat the disease. In efforts to evaluate potential therapeutic candidates, a cytotoxic glucose analog, streptozotocin (STZ), has been widely employed to induce diabetes in small and large animal models. Despite its wide use, the effects of STZ treatment on pancreatic insulin-producing β-cells, particularly on gene expression, remain largely unknown. Another compound that is widely used in diabetes research is glucagon-like peptide-1 (GLP-1), a multifunctional incretin hormone that inhibits glucagon secretion, induces glucose-responsive insulin secretion from β-cells, inhibits β-cell apoptosis and stimulates the proliferation of β-cells. GLP-1 receptor agonists and inhibitors for GLP-1 degradation have been used successfully to treat type 2 diabetes; however, recent reports suggest an increased risk of pancreatitis and pancreatic cancer in patients chronically treated with some of these drugs. To devise strategies to overcome the associated toxicities, it is important to fully understand the pathways affected by the long-term administration of GLP-1 analogs *in vivo*.

**Results**

Here, the authors analyzed the effects of STZ-induced diabetes and GLP-1-mediated β-cell protection in STZ-treated mice on the genome-wide expression patterns in pancreatic islets. They report that STZ administration induces p53-responsive genes in affected islets, including *p21*, which encodes a mediator of cell cycle arrest. STZ treatment is also shown to suppress the expression of an array of genes linked to key β-cell functions or progression of diabetes development, suggesting the presence of global β-cell defects in STZ-treated islets. The group identified potential diabetes-associated genes that are highly expressed in untreated islets and strongly suppressed by STZ, including *Tmem229B*, *Prss53* and *Ttc28*. Sustained overexpression of GLP-1 from a pancreas-targeted AAV vector protected mice from STZ-induced diabetes through the preservation of β-cell mass. Pancreatic GLP-1 expression strongly upregulated selected genes (e.g. *Cxcl13*, which has pro-β-cell survival effects) in STZ-damaged islets but not in islets that were not treated with STZ. The protective effects of GLP-1 overexpression lasted for 2 months and, unexpectedly, did not influence STZ-imposed effects on global gene expression.

**Implications and future directions**

The fundamental goal of diabetes research is to determine why β-cells lose their functionality in individuals with diabetes. The data presented here suggest that STZ induces a cellular stress response through p53 activation, which turns off a wide range of genes that are essential for β-cell functions. Further analysis of this process could provide essential insights into the development of diabetes in humans. Additionally, the study demonstrates the utility of *Glp-1* gene therapy to prevent β-cell loss and induce the expression of selected genes, such as *Cxcl13*, that promote β-cell survival in damaged islets. Further studies could reveal the potential roles of the identified genes in β-cell protection. The long-term *Glp-1* gene-therapy strategy described in this study provides a unique platform to study the potential adverse effects of chronic GLP-1 treatment in rodents; these findings could then be extended to humans.

## RESULTS

### Development of pancreas-targeting AAV vectors

**Fig. 1.**
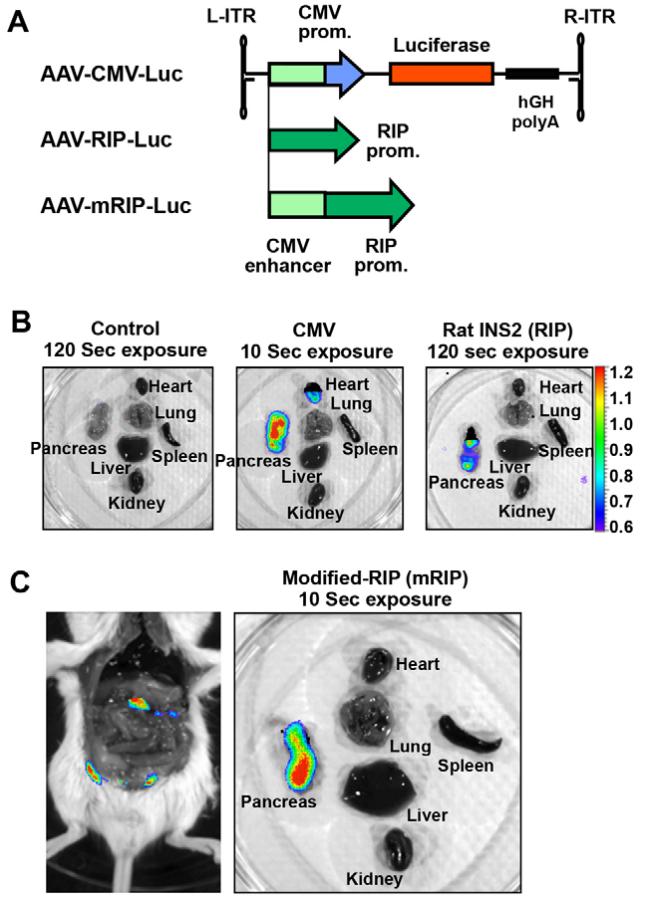
**AAV9-vector-mediated pancreatic gene delivery.** (A) Schematic representation of AAV vectors with different internal promoters. The AAV vector contained either cytomegalovirus IE (CMV), rat insulin promoter (RIP) or modified RIP promoter (mRIP). mRIP contains the CMV enhancer sequence in front of the RIP promoter. (B) *In vivo* transgene expression in mice systemically administrated with the AAV9 vectors. Control mice showed no luciferase signal. Mice receiving AAV9-CMV-*Luc* showed strong signals in heart and pancreas at 10 seconds of exposure. Mice receiving AAV9-RIP-*Luc* showed a weak pancreas-specific signal after a 120-second exposure. (C) Mice receiving AAV9-mRIP-*Luc* showed strong signals in pancreas at 10 seconds of exposure.

The AAV9 vector is known to possess a natural cardiotropic phenotype. We found that intraperitoneal administration of Balb/c mice with an AAV9 vector encoding firefly luciferase under the control of a CMV promoter (Fig. 1A) led to predominant transduction of the pancreas as well as the heart (Fig. 1B). To restrict transgene expression to the pancreas, we generated pAAV-RIP-*Luc*, which harbors a rat insulin promoter (RIP) instead of the constitutively active CMV promoter (Fig. 1A). Mice administered with the AAV-RIP-*Luc* vector demonstrated pancreas-specific luciferase expression. However, the luciferase expression from the RIP promoter was considerably weaker than those from the CMV promoter, and a longer exposure time was necessary to detect comparable signals from mice injected with the AAV-RIP-*Luc* vector (120 seconds for AAV-RIP-*Luc* versus 10 seconds for AAV-CMV-Luc) (Fig. 1B). To increase transgene expression, we generated the AAV-mRIP-*Luc* vector with a modified RIP (mRIP) promoter, which has the CMV enhancer sequence upstream of the RIP promoter (Fig. 1A). The mRIP vector demonstrated improved transgene expression, while maintaining the pancreas-targeted phenotype upon intraperitoneal administration (Fig. 1C).

### Pancreatic expression of REG3B–GLP-1 prevented STZ-induced hyperglycemia

**Fig. 2.**
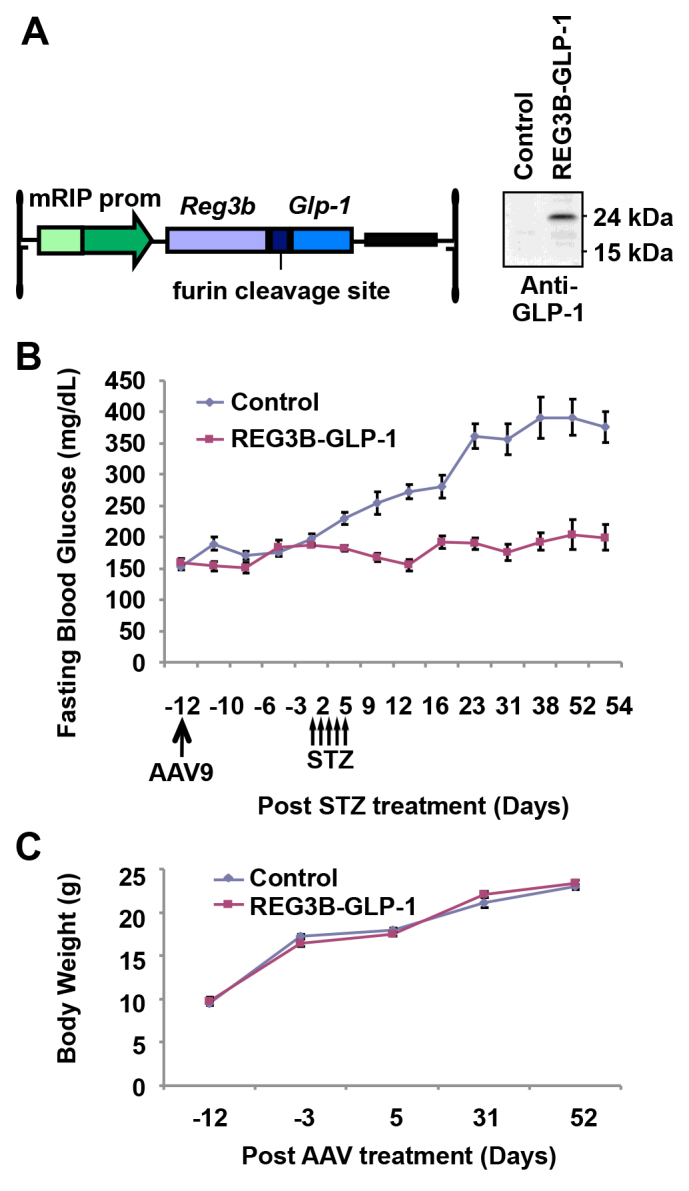
**Pancreas-specific gene delivery of *Reg3b–Glp-1* protects mice from STZ-induced diabetes.** (A) The AAV vector construct was designed to express the REG3B–GLP-1 fusion protein conjugated with the ubiquitous furin cleavage site under the control of the mRIP promoter. GLP-1 expression was confirmed by western blotting analysis with anti-GLP-1 antibody. (B) Fasting blood glucose levels of mice receiving PBS (control, *n*=8) or AAV9 mRIP *Reg3b–Glp-1* (REG3B-GLP-1, *n*=8) vectors at day −12. Mice received five consecutive low-dose intraperitoneal injections of STZ at days 1–5. Error bars represent s.e.m. (C) Average group body weight of control and REG3B–GLP-1 mice. Error bars represent s.e.m.

GLP-1 is known to have β-cell protective effects. REG3 proteins, a family of secreted C-type lectins, are implicated in β-cell regeneration. To achieve pancreatic overexpression of GLP-1 without expressing glucagon, we designed a codon-optimized sequence that encodes an artificial REG3B–GLP-1 fusion protein linked by a furin cleavage sequence (Fig. 2A). This *Reg3b–Glp-1* sequence was then cloned into the pancreas-targeted mRIP AAV vector. Expression of the REG3B–GLP-1 fusion protein was verified by immunoblotting using anti-GLP-1 antibody. We then assessed the β-cell-trophic and/or protective effects of pancreatic REG3B–GLP-1 expression using the STZ-induced diabetes model of mice. C57BL/6 mice were intraperitoneally injected with the *Reg3b–Glp-1* vector. At 2 weeks after vector administration, treated and control mice received five consecutive days of intraperitoneal administrations of low-dose STZ (50 mg/kg body weight). Their blood glucose levels and body weights were monitored for 2 months. The vector-pretreated mice remained normoglycemic, whereas control mice became hyperglycemic upon STZ treatment (Fig. 2B). No change in body weight was observed between groups (Fig. 2C).

### Pancreatic REG3B–GLP-1 expression preserved insulin-positive β-cells upon STZ treatment

**Fig. 3.**
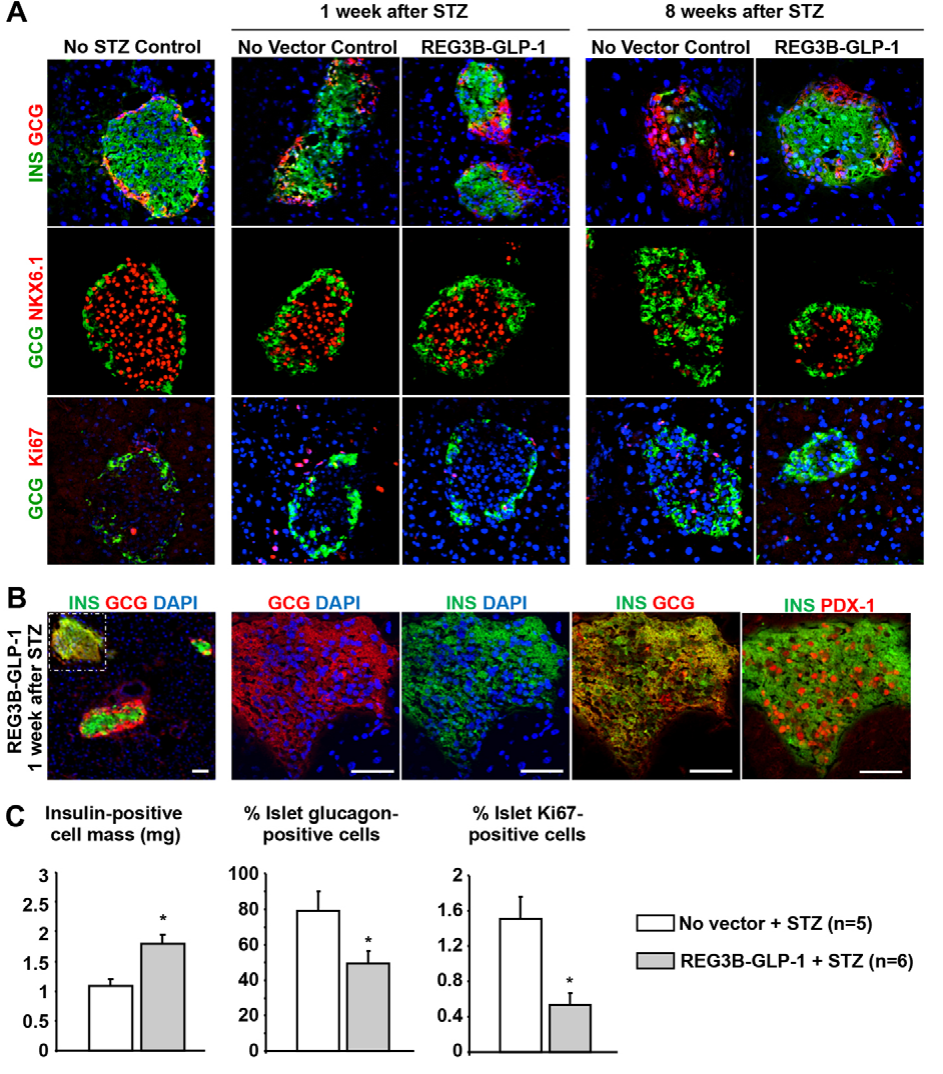
**REG3B–GLP-1-treated pancreatic islets maintain insulin-positive cells after STZ induction.** (A) Immunohistochemistry showing islet structure without STZ and REG3B–GLP-1 (left column), 1-week post STZ induction with or without REG3B–GLP-1 therapy (middle columns), and 8-weeks post STZ induction with or without REG3B–GLP-1 therapy (right columns). Insulin and glucagon (top row), glucagon and Nkx6.1 (middle row), and glucagon and ki67 (bottom row) are shown. All images were taken with 40× objective. (B) Immunostaining of islets 1-week post STZ induction with AAV9 mRIP *Reg3b–Glp-1* delivery. A small population of islets was found to be bi-hormonal for glucagon and insulin. The left panel is 10× objective of pancreas cross-section, with the islet with insulin and glucagon dual-positive cells indicated by a dashed box. Other panels show serial sections of a bi-hormonal islet, stained with combinations of anti-insulin, -glucagon or -Pdx1 antibodies, at higher magnifications (40× objective). Nuclei were counterstained by DAPI. Scale bars: 50 μm. (C) 8 weeks after STZ induction insulin-positive cell masses were determined as described in the Materials and Methods (left panel). Percent of total glucagon area to insulin area was determined from five random islets per individual (middle panel). Percent islet cell proliferation was determined by counting the number of ki67-positive cells within insulin- and glucagon-positive regions of five random islets (right panel).

To assess the underlying mechanism of the REG3B–GLP-1 therapy, we analyzed the influence of low-dose STZ treatments on pancreatic islets. Immunohistochemical analysis of pancreatic sections revealed that the expression of insulin in Nkx6.1-positive β-cells was impaired in control mice at 1 week after initiation of STZ treatment (Fig. 3A). In contrast, mice pretreated with the *Reg3b–Glp-1* vector generally showed apparently healthy islets with sustained insulin-positive β-cells (Fig. 3A,B). In rare occurrences, REG3B–GLP-1 STZ-induced mice had islets that comprised insulin and glucagon dual positive cells (Fig. 3B). These cells also expressed nuclear Pdx-1, a transcription factor found in mature β-cells (Fig. 3B). STZ or REG3B–GLP-1 treatments showed no notable effects on glucagon-positive α-cells at 1 week post STZ treatment (Fig. 3A).

At 8 weeks after STZ treatment, marked reductions in Nkx6.1-and insulin-positive β-cells and a predominance of α-cells were evident in the islets of STZ-treated mice (Fig. 3A). Pancreatic REG3B–GLP-1 expression led to preservation of insulin- and Nkx6.1-expressing β-cells after STZ treatment. Insulin-positive cell mass was significantly higher in the STZ REG3B–GLP-1-treated mice than the STZ non-vector-treated control mice (Fig. 3C). A higher glucagon-positive cell mass was observed in the STZ-treated control group. Although GLP-1 is known to increase β-cell mass *in vivo*, pancreatic REG3B–GLP-1 expression did not accelerate β-cell proliferation after STZ treatment.

### Pancreatic expression of GLP-1, but not REG3B, was responsible for β-cell protection

**Fig. 4.**
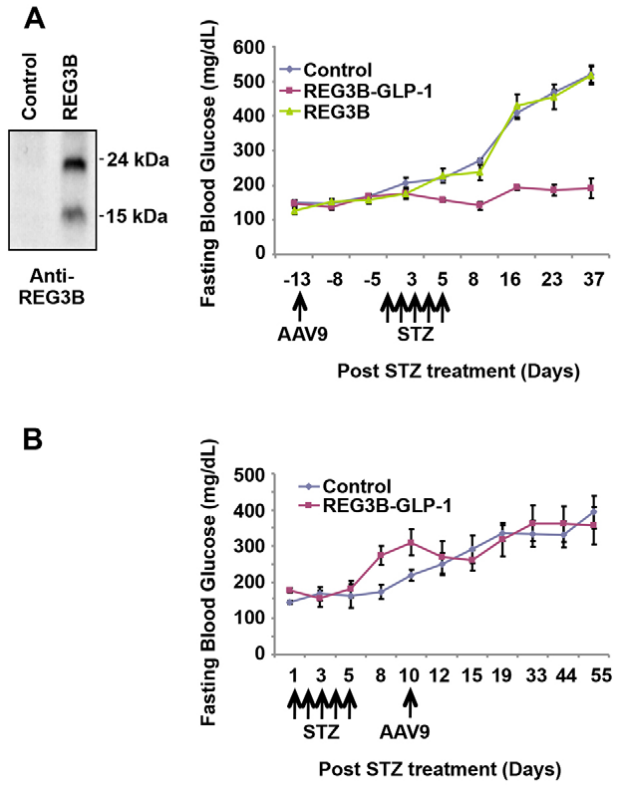
**Therapy of REG3B–GLP-1 vector requires GLP-1 to maintain normoglycemia.** (A) We generated an AAV9 vector expressing REG3B alone (AAV9 mRIP *Reg3b*). REG3B expression was verified by western blotting (left panel). Fasting blood glucose levels among mice administered PBS (control), AAV9 mRIP *Reg3b–Glp-1* vector, and AAV9 mRIP *Reg3b* vector. AAV9 vector was administered 2 weeks prior to five consecutive STZ administrations (*n*=5/group), and the blood glucose levels were monitored. (B) The AAV9 mRIP *Reg3b–Glp-1* vector was unable to reverse STZ-induced diabetes. Five low doses of STZ were administered prior to AAV9-mediated therapy (*n*=3/group). Error bars represent s.e.m.

To assess the contributions of GLP-1 and REG3B expression on the β-cell protection from low-dose STZ treatments, we generated and tested an AAV9 vector that carries only *Reg3b* cDNA. Expression of the pAAV-*Reg3b* vector was verified by immunoblotting using anti-REG3B antibody (Fig. 4A, left panel). Intraperitoneal administration of the AAV9 vectors, followed by STZ challenge, demonstrated clear therapy in AAV9–*Reg3b–Glp-1*-treated mice (Fig. 4A). However, no notable effect was observed in the mice treated with the AAV9-*Reg3b* vector, indicating that pancreas-targeted expression of GLP-1, but not REG3B, prevented the STZ-induced β-cell loss. We also tested the effects of pancreatic REG3B–GLP-1 expression on the reversal of STZ-induced hyperglycemia. Mice were pre-treated with multiple low doses of STZ, followed by intraperitoneal administration of the REG3B–GLP-1-expressing vector, and the blood glucose levels were monitored for 6 weeks after vector administration. As shown in Fig. 4B, no reversal effects were observed in any of the treated mice upon vector administration.

### Short-term, low-dose STZ treatment induced p53-responsive genes and suppressed a wide range of genes associated with β-cell functions in islets

**Fig. 5.**
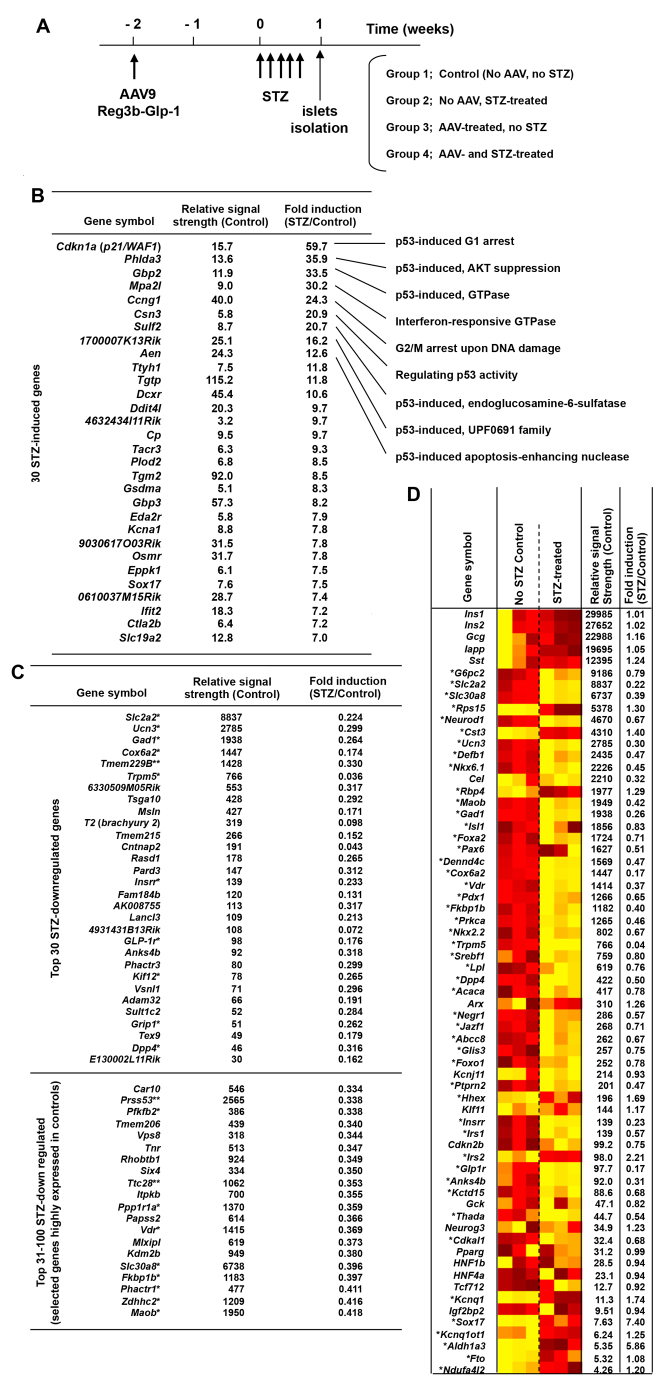
**The global gene expression profiles of purified islets from mice that received STZ.** (A) Schematic diagram of the experimental set-up. Mice were transduced with AAV9 mRIP–*Reg3b–Glp-1* 2 weeks before STZ administration. Islets were then isolated 7 days later (*n*=3/group). (B) Top 30 STZ-induced genes are indicated with fold increase over control. (C) Top 30 STZ downregulated genes. A single asterisk (*) indicates genes linked to diabetes or β-cell functions. A double asterisk (**) indicates genes with >1000 signal strengths, strongly suppressed by STZ, and with no known link with diabetes or β-cell functions. (D) Heatmap analysis of islet-specific genes for normal mice (control) and STZ treatments. A single asterisk (*) indicates genes linked to diabetes or β-cell functions. Gene expressions are compared based on varying degrees of yellow (lower) to red (higher). Within the control column of the heatmap, shades of yellow or red refer to normal expression levels, whereas in the STZ-treated column shades of yellow or red refer to lower or higher gene expressions compared with the controls.

For a better understanding of the influences of STZ treatment and pancreatic *Glp-1* gene therapy on the integrity of β-cells, we determined the global gene expression profiles of islets from mice that received AAV9–*Reg3b–Glp-1* and/or STZ administrations (Fig. 5A). To minimize the secondary effects caused by hyperglycemia, we isolated islets at 1 week after the initial STZ treatment. At this time point, STZ-treated islets still maintained typical islet morphologies with Nkx6.1-positive β-cells, although the insulin expression was notably reduced (Fig. 3A). Comparison of the transcriptome of islets of control and STZ-treated mice identified STZ-induced genes (Fig. 5B). In accordance with the reported genotoxicity of STZ, many STZ-induced genes were p53-responsive. For instance, STZ administration resulted in a 60-fold increase in the transcripts of *p21* (also known as cyclin-dependent kinase inhibitor *CDKN1A*, *Waf1* and *Cip1*), a well-known mediator of p53-dependent cell cycle arrest (Fig. 5B). It is likely that low-dose STZ administrations induce DNA damage in β-cells, which activates p53 and a series of p53-responsive, senescence-regulating and apoptosis-regulating genes. Transcriptome analysis also identified genes that are strongly downregulated by low-dose STZ treatments. Intriguingly, most of the identified genes, which showed strong signals in normal islets but were potently suppressed by STZ, were previously linked with important β-cell functions or diabetes development, such as *Slc2a2*, *Ucn3*, *Gad1*, *Cox6a2*, *Trpm6* and *Vdr* (Fig. 5C). The heat map analysis of selected genes, which were previously linked with β-cell functions or diabetes, further demonstrated STZ-mediated suppression of a wide range of β-cell-and/or diabetes-related genes, including *Slc30a8*, *Neurod1*, *Nkx6.1*, *Isl1*, *Foxa2* (*Hnf3b*), *Pax6*, *Pdx1*, *Fkbp1b*, *Prkca*, *Dpp4*, *Abcc8* and *Foxo1* (Fig. 5D). These data indicate that short-term, low-dose STZ administration not only induces DNA damage and p53, but also affects global β-cell functions. Of note, the *Tmem229B*, *Prss53* and *Ttc28* genes, which have not been linked to β-cell functions or diabetes, showed strong signals in untreated islets but were strongly suppressed by STZ (Fig. 5C). It is plausible that those three genes also play substantial roles in β-cells.

### Identification of genes induced by pancreatic REG3B–GLP-1 overexpression and STZ-induced β-cell damage

**Fig. 6.**
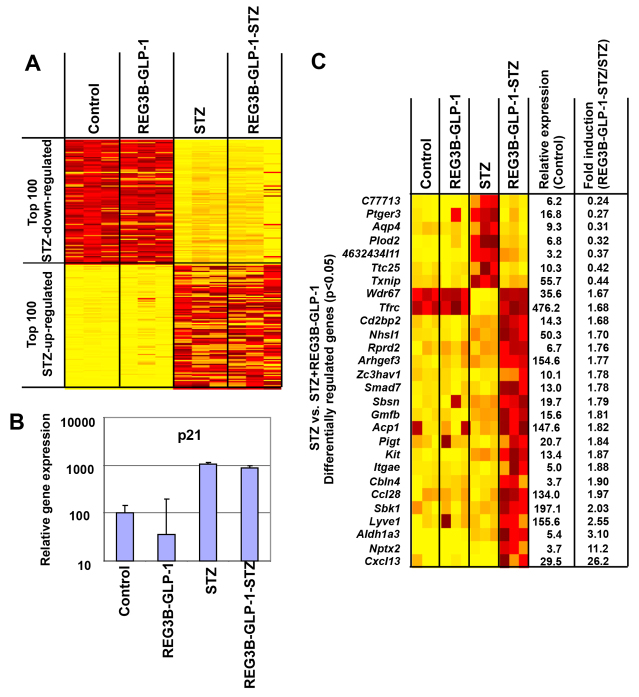
**Transcriptome profile of purified mouse islets from mice administered AAV9–*Reg3b–Glp-1* vector and/or STZ.** (A) Heatmap depicting the top 100 up- and downregulated genes among the four treatments. Gene expressions are displayed by brightness of yellow (downregulated) or red (upregulated) as compared with the controls. (B) qPCR confirmation of *p21* relative gene expressions based on transcriptome data. Error bar represents s.e.m. (*n*=3). (C) Heatmap showing differentially expressed genes between mice treated with STZ alone or STZ and AAV9 –*Reg3b–Glp-1*. Gene expressions are displayed by brightness of yellow (downregulated) or red (upregulated) as compared with the controls.

Next, we determined the influence of pancreatic overexpression of REG3B–GLP-1 on the islet transcriptome. Despite its potent β-cell protective effects, pancreatic GLP-1 expression showed little effects on the islet transcriptome. In general, GLP-1 expression did not block the STZ-imposed changes in the transcriptome (Fig. 6A). For instance, induction of the p53-responsive p21 was not significantly blocked (Fig. 6B), suggesting that GLP-1 does not prevent STZ-induced DNA damage and subsequent p53 induction. Network analysis was then performed to identify the pathways that are differentially expressed between the GLP-1/STZ and STZ only groups. The MetaCore analysis using the ‘Analyze Networks’ algorithm using the default parameter settings identified the apoptosis and programmed cell death pathways as the most relevant network (supplementary material Fig. S1), which confirmed previous studies demonstrating GLP-1-mediated β-cell survival. Further bioinformatic analysis identified genes that were differentially expressed between the GLP-1/STZ and STZ groups (Fig. 6C). Those genes were separated into three categories; (i) strongly induced by STZ but pancreatic GLP-1 expression blocks their induction (e.g. *Ptger3*), (ii) suppressed by STZ but pancreatic GLP-1 expression blocks their suppression (e.g. *Wdr67*), and (iii) not induced by GLP-1 or STZ only, but strongly induced in the mice treated with both GLP-1 and STZ (e.g. *Cxcl13* and *Nptx2*).

## DISCUSSION

Here we studied the influence of multiple low-dose STZ treatments and pancreatic *Reg3b–Glp-1* gene therapy on the genome-wide gene expression profiles of pancreatic islets. Our results demonstrated induction of p53-responsive genes and suppression of a wide range of diabetes-related genes upon STZ treatment. Pancreatic overexpression of REG3B–GLP-1 prevented the β-cell loss upon STZ challenge and protected mice from developing hyperglycemia. Despite the notable β-cell protective effect, pancreatic REG3B–GLP-1 overexpression did not block STZ-imposed changes in the gene expression profiles of mouse islets. Instead, pancreatic REG3B–GLP-1 expression suppressed the apoptotic pathway and induced selected genes in STZ-damaged islets.

STZ has been widely used to induce experimental diabetes in animals. Upon systemic delivery, STZ is taken up through glucose transporter Glut2, then induces DNA damage through alkylation and fragmentation of DNA (Morgan et al., 1994; Delaney et al., 1995). As shown in Fig. 3, low doses of STZ treatment did not immediately kill β-cells *in vivo*. Many β-cells retained nuclear Nkx6.1 signals at 1 week after the initiation of STZ administration, although insulin expression was significantly impaired in the treated cells. Our genome-wide gene expression profiling identified notable upregulation of a series of p53-responsive genes, including a master regulator of growth arrest and cellular senescence, p21. Many other factors are also known to induce cell cycle arrest and cellular senescence. Because p53 is activated by DNA damage, our finding is in accordance with the reported genotoxicity of STZ. It is also notable that most of the genes identified as ‘highly downregulated by STZ’ play important roles in β-cell functions or β-cell development, but are not house-keeping genes (Fig. 5). This selective suppression of a wide range of β-cell-related genes is probably an underlying mechanism for the β-cell failure in mice shortly after low-dose STZ treatment. At present, it remains to be determined how STZ can selectively suppress those β-cell-related genes. In human type 2 diabetes, glucolipotoxicity, ER stress and oxidative stress have been implicated in the progressive β-cell failure (Prentki and Nolan, 2006). Because STZ is reported to generate reactive oxygen species that show antineoplastic properties (for a review, see Bolzán and Bianchi, 2002), we postulate that STZ-induced oxidative stress plays a role in suppressing the expression of genes that are crucial for normal β-cell functionality. In summary, our data and a study from Zheng et al., which showed that p53-deficient mice were more susceptible to STZ-induced diabetes (Zheng et al., 2005), suggest that the flow of the STZ-mediated β-cell failure is as follows: (i) low-dose STZ administration induces modest DNA damage, (ii) modest DNA damage does not kill cells but activates p53, (iii) activated p53 induces a series of p53-dependent genes, (iv) p53-induced factors arrest β-cell proliferation, and (v) STZ-induced stress turns off a wide range of genes that are essential for β-cell functions.

As depicted in Fig. 2B, pancreatic expression of REG3B–GLP-1 reproducibly prevented STZ-induced hyperglycemia in all mice tested. However, pancreatic overexpression of REG3B alone showed no β-cell protective effect (Fig. 4A), indicating that GLP-1, but not REG3B, played an essential role in protecting mice from STZ-induced hyperglycemia. An immunohistochemistry study has demonstrated that *Glp-1* gene therapy protected β-cells from STZ-induced damage (Fig. 3A), resulting in higher β-cell mass than in untreated controls at 2 months after STZ treatment (Fig. 3B). Transcriptome analysis also found activation of genes that are linked to β-cell survival (supplementary material Fig. S1), which confirmed previous studies (Li et al., 2003; Yusta et al., 2006). Although GLP-1 therapies have shown increased β-cell proliferation (Edvell and Lindström, 1999; Liu and Habener, 2008; Gaddy et al., 2010), we did not see a significant increase in β-cell proliferation upon *Glp-1* gene therapy. Additionally, we were unable to reverse STZ-induced diabetes. Those observations indicate that the primary mechanism of *Reg3b–Glp-1* gene therapy is due to GLP-1-mediated β-cell survival, but not enhanced β-cell regeneration or neogenesis. It is notable that similar results have been reported in a transgenic mouse model that chronically expresses a GLP-1 analog, exendin 4, which demonstrated normal β-cell mass and islet histology (Stoffers et al., 2000). We postulate that sustained GLP-1 expression from β-cells might downregulate the GLP-1 receptor signaling pathway for β-cell proliferation.

In summary, our study demonstrated promising, long-term β-cell-protective effects through pancreas-targeted *Reg3b–Glp-1* gene therapy. Because recent reports demonstrated the increased risks of pancreatitis and pancreatic cancer in patients chronically treated with GLP-1 analogs and DPP4 inhibitors (Gier et al., 2012), it would be necessary to monitor the potential toxicity of long-term pancreatic GLP-1 overexpression on pancreatitis and pancreatic cancers in mice. We are also interested in the effects of pancreatic GLP-1 expression. Previous studies have found increased expression of prohormone converting enzymes in α-cells, which leads to an increase in GLP-1 production, enhancing β-cell growth and survivability (Nie et al., 2000; Yano et al., 2007). Notably, damaged β-cells are reported to produce *Cxcl12* (*Sdf1*) to induce GLP-1 production in adjacent α-cells (Yano et al., 2007). Our transcriptome analysis identified the upregulation of *Cxcl13* in STZ-damaged islets upon *Glp-1* gene therapy. Intriguingly, *Glp-1* gene therapy or STZ treatment alone did not increase the expression of *Cxcl13*, suggesting that *Cxcl13* is induced in response to β-cell damage and GLP-1 treatment. Given the pro-β-cell-survival effects of *Cxcl12* in inducing GLP-1 production in α-cells (Yano et al., 2007), GLP-1-induced *Cxcl13* production in damaged β-cells might also play a β-cell protective role.

## MATERIALS AND METHODS

### Mice

All studies were approved by Mayo Clinic Institutional Animal Care and Use Committee. All experimental mice, 5-week-old C57BL/6J, were purchased from Jackson Laboratories. Mice were maintained under a 12-hour light-dark cycle and were provided with irradiated Rodent Laboratory Chow (Purina 5053) and water *ad libitum*. To induce diabetes, mice received five consecutive intraperitoneal injections of STZ (50 mg STZ/kg body weight) resuspended in 0.1 M citrate buffer (pH 4.5) over the course of 5 days. Fasting (4–6 hours) blood glucose levels were monitored weekly or bi-weekly by FreeStyle Lite Blood Glucose Monitor (Abbott Laboratories, Abbott Park, IL).

### Cells

HEK 293T cells were maintained in Dulbecco’s modified Eagle’s medium supplemented with 10% calf serum, 50 U/ml penicillin and 50 μg/ml streptomycin. Cells were kept at 37°C with 5% CO_2_.

### Plasmids

The pAAV-CMV-*Luc* vector genome construct, which drives firefly luciferase expression by a CMV internal promoter, was described previously (Cataliotti et al., 2011). The 721 bp rat insulin promoter (RIP) fragment was amplified by PCR primers Forward Mlu1 5′-GCAT**ACGCGT**ATCGATAACCACTCCAAGTGGA-3′ and Reverse BamH1 5′-GCGTAAGCTTAGGCCT**GGATCC**TTA-3′ (bold indicates restriction sites *Mlu*1 or *Bam*H1). pAAV-RIP-*Luc* vector construct was generated by replacing the CMV promoter of pAAV-CMV-*Luc* with the RIP sequence using unique *Mlu*I and *Bam*HI sites. The AAV vector with a modified RIP promoter, pAAV-mRIP-*Luc*, was generated by introducing the sequence containing CMV enhancers, which was amplified by PCR with primers Forward Mlu1 5′-GCTC**ACGCGT**GTTGACATTGATTATTGACTAGTTATTAAT-3′ and Reverse Mlu1 5′-GCTC**ACGCGT**TAGACCTAGGGTAAAGATTATTAACAAGGGGCCAGATGGCCTGATGAAGCCCACCGTACACGCCTACCGCCCAT-3′ into the *Mlu*I site of pAAV-RIP-*Luc*. The codon-optimized nucleotide sequence encoding the murine REG3B–GLP-1 fusion protein, linked with a furin cleavage site, was synthesized by GenScript, and cloned into the place of the luciferase gene in pAAV-mRIP-*Luc* with the unique *Bam*HI and *Xho*I sites.

### AAV9 vectors

The AAV9 vector stocks were produced in human 293T cells using the helper-free transfection method according to the manufacturer’s protocol (Stratagene, Santa Clara, CA). Specifically, we used AAV9 capsid-expressing plasmid pRep2Cap9 (kindly provided by Dr James Wilson, University of Pennsylvania, PA). Firefly luciferase-or mouse REG3B–GLP-1 fusion-protein-encoding AAV genome constructs were packaged. At 3 days after transfection, AAV9 vector-producing 293T cells were harvested for vector purification. The cells were lysed by freeze and thaw cycling, followed by ultracentrifuge concentration (∼400,000 ***g*** for 2 hours) through Optiprep Density Gradient Medium (Sigma, St Louis, MO). The resulting AAV9 vectors were desalted and concentrated using Amicon Ultra-15 100k filtration (Amicon, Billerica, MA) before being resuspended in PBS. The titers (genomic copy numbers/ml) of concentrated AAV9 vector stocks were determined by quantitative PCR using plasmid DNA standards and AAV genomic sequence-specific primers and fluorescent probe.

### STZ and AAV vector administrations

In preventative diabetes experiments, mice first received an intraperitoneal injection of AAV9 vectors at a final dose of 1×10^11^ genome copies (gc)/mouse. Two weeks later the mice received 50 mg STZ/kg body weight intraperitoneally over the course of five consecutive days. In reversal diabetes experiments, mice first received 50 mg STZ/kg body weight intraperitoneally over the course of five consecutive days. Once diabetes was established by obtaining two consecutive blood glucose readings of >250 mg/dl, mice received AAV9 vectors intraperiteonally at 1×10^11^ gc/mouse.

### Immunoblotting

To verify the expression of the REG3B–GLP-1 fusion protein from the AAV9 vector, 293T cells were transfected with either pAAV–mRIP–*Reg3b–Glp-1* or pAAV-mRIP-*Reg3b* plasmid. At 3 days after transfection, cell media and cell lysates were harvested. Cells were lysed in RIPA buffer containing 50 μM DPP-IV inhibitor (EMD Millipore Corporation, Billerica, MA) in addition to protease inhibitor cocktail (Roche, Indianapolis, IN). Cell media was collected, passed through a 45-μm filter and mixed with DPP-IV inhibitor at 50-μM concentration. SDS-PAGE was conducted with 15% Tris-glycine gel (Bio-Rad, Hercules, CA). Immunoreactive REG3B–GLP-1 fusion protein and REG3B was detected using rabbit anti-GLP-1 antibody (1:200, Epitomics, Burlingame, CA) and rat anti-REG3B antibody (1:1000, R&D Systems, Minneapolis, MN), respectively. HRP-conjugated anti-rabbit or anti-rat antibodies (1:2000, Jackson ImmunoResearch Laboratories, Inc., West Grove, PA) were used for visualization.

### Immunostaining

Tissues were embedded and frozen in OCT Compound (Sakura Finetek USA, Inc., Torrance, CA). 7-μm pancreatic cryosections were obtained and immediately fixed in 4% paraformaldehyde for 30 minutes at room temperature. The sections were then permeabilized with PBS containing 0.3% Triton-X for 10 minutes, washed and then blocked for 1-hour in PBS containing 5% FBS at room temperature. Primary antibody treatment was done overnight (∼14 hours) in humidity chambers to prevent drying. Sections were washed three times with PBS and then incubated with species-appropriate Alexa-Fluor-488- or 594-IgG (H^+^L) (Invitrogen, Grand Island, NY) for 60 minutes in the presence of DAPI. Cells were then observed with a Zeiss LSM 510 confocal laser scanning microscope and the images were analyzed with Zeiss imaging software. Antibodies and concentrations used for immunocytochemistry include: guinea pig anti-insulin (1:400, Dako Corporation, Carpinteria, CA), mouse anti-insulin (1:300, Sigma-Aldrich, St Louis, MO), mouse monoclonal to glucagon (1:300, Abcam, Cambridge, MA), rabbit polyclonal to Pdx-1 (1:100, Abcam, Cambridge, MA), goat polyclonal against Nkx6.1 (1:200, R&D Systems, Minneapolis, MN), rabbit polyclonal to ki67 (1:100, Abcam, Cambridge, MA), and goat polyclonal to Cxcl13 (1:200, R&D Systems, Minneapolis, MN).

### Insulin-positive mass analysis

Three 7-μm thick whole pancreatic sections (200-μm depths) were collected per animal and counterstained for anti-insulin and anti-glucagon with DAPI labeling. Insulin-positive mass was determined by measuring the relative insulin-positive tissue area to total tissue area with KS400 Image Analysis Software (Version 3.0, Zeiss, Jena, Germany). Insulin-positive cell mass was determined using the formula: insulin cell mass (mg)=insulin-positive area/total tissue area × total pancreatic weight (mg).

### Mouse islet isolation and RNA extraction

Murine islets were isolated using a modified version of a previously published islet isolation protocol (Rostambeigi et al., 2011). Briefly, islets were isolated using a discontinuous Dextran-based gradient. Islets were handpicked under magnification using a modified 200-μl pipette tip attached to a threaded syringe (Hamilton Company, Reno, NV). Heterogeneity of islets was not directly assessed after isolation. Total RNA was extracted by Qiagen RNeasy Micro Kit (Qiagen, Gaithersburg, MD).

### Microarray

Microarray analysis was performed using the Affymetrix HG-U133 Plus2 GeneChip Array platform (Affymetrix, Santa Clara, CA), and data were analyzed as reported previously (Thatava et al., 2013). Student’s *t*-test was performed to analyze the significance of the changes (*P*<0.05) in the normalized gene expression levels between the four groups (control, STZ only, REG3B–GLP-1 only, REG3B–GLP-1 with STZ). Heatmap Builder software (kindly provided by Dr Euan Ashley, Stanford School of Medicine, CA) was used to generate the heatmap for the transcriptome data set.

### Reverse transcription polymerase chain reaction (RT-PCR)

RNA from pancreatic sections was isolated using the RNeasy Mini Kit (Qiagen, Gaithersburg, MD). Random-primed RNA was reverse-transcribed using superscript III (Invitrogen, Grand Island, NY). Zymo Taq DNA polymerase was used to detect the *p21* and *Glp-1r* transcripts.

## Supplementary Material

Supplementary Material
