# Supplementary material for: Global gene expression profiling of pancreatic islets in mice during streptozotocin-induced β-cell damage and pancreatic Glp-1 gene therapy
Source: Dis Model Mech. 2013 Jul 4;6(5):1236–45. doi: 10.1242/dmm.012591 (PMC3759343; doi:10.1242/dmm.012591)
Supplement: Supplementary Material [file supp_6_5_1236__index.html]

Global gene expression profiling of pancreatic islets in mice during streptozotocin-induced β-cell damage and pancreatic Glp-1 gene therapy — Global gene expression profiling of pancreatic islets in mice during streptozotocin-induced β-cell damage and pancreatic Glp-1 gene therapy — Supplementary Material 

# Global gene expression profiling of pancreatic islets in mice during streptozotocin-induced β-cell damage and pancreatic *Glp-1* gene therapy

## DMM012591 Supplementary Material

**Files in this Data Supplement:**

- **Supplementary Material PDF**
