# Supplementary material for: Global gene expression profiling of pancreatic islets in mice during streptozotocin-induced β-cell damage and pancreatic Glp-1 gene therapy
Source: Dis Model Mech. 2013 Jul 4;6(5):1236–45. doi: 10.1242/dmm.012591 (PMC3759343; doi:10.1242/dmm.012591)
Supplement: Supplementary Material [file supp_012591_DMM012591.pdf]

Supplementary Fig. 1   Tonne et al.

Network analysis identified the pathways related to apoptotic and programmed cell death processes as the most relevant network affected in islets upon Glp-1 gene therapy.

Most relevant networks

The gene content of the uploaded files is used as the input list for generation of biological networks using Analyze Networks (AN) algorithm with default settings. This is a variant of the shortest paths algorithm with main parameters of 1. relative enrichment with the uploaded data, and 2. relative saturation of networks with canonical pathways. These networks are built on the fly and unique for the uploaded data. In this workflow the networks are prioritized based on the number of fragments of canonical pathways on the network.

No	Processes	Size	Target	Pathways	zScore	gScore
1	apoptotic process (76.6%), programmed cell death (76.6%), regulation of apoptotic process (83.0%), regulation of programmed cell death (83.0%), regulation of cell death (83.0%)	52	8	189	7.68	243.93
2	transmembrane receptor protein tyrosine kinase signaling pathway (76.0%), enzyme linked receptor protein signaling pathway (80.0%), neuron projection development (72.0%), neuron development (74.0%), neuron projection morphogenesis (68.0%)	50	9	52	8.87	73.87

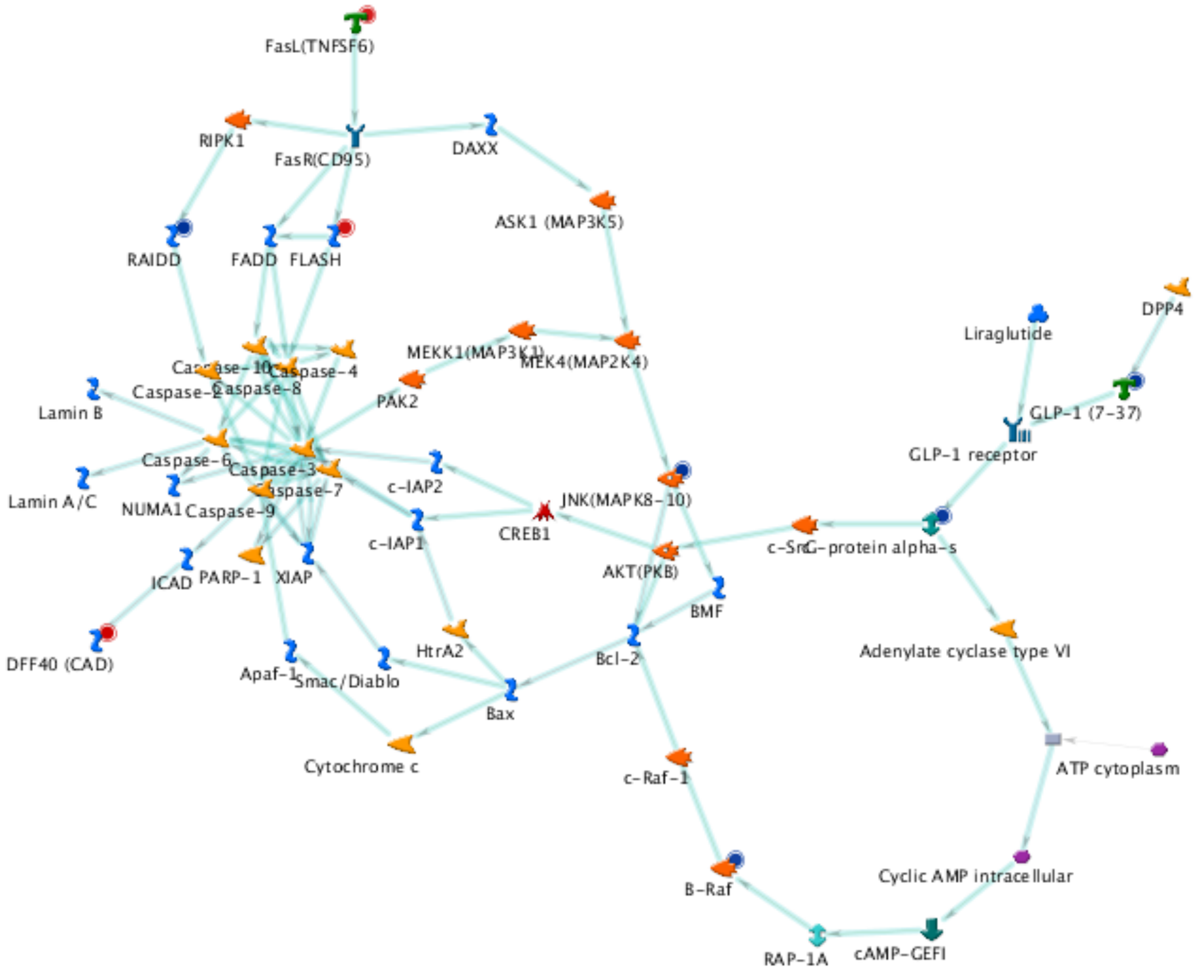

The top scored (by the number of pathways) AN network from cmp3_p05. Thick cyan lines indicate the fragments of canonical pathways. Up-regulated genes are marked with red circles; down-regulated with blue circles. The 'checkerboard' color indicates mixed expression for the gene between files or between multiple tags for the same gene.
